# Building Positive Organizations: A Typology of Positive Psychology Interventions

**DOI:** 10.3389/fpsyg.2021.769782

**Published:** 2021-11-16

**Authors:** Marianne van Woerkom

**Affiliations:** ^1^Department of Human Resource Studies, Tilburg University, Tilburg, Netherlands; ^2^Center of Excellence for Positive Organizational Psychology, Erasmus University Rotterdam, Rotterdam, Netherlands

**Keywords:** positive psychology, positive psychology intervention (PPI), employee well-being, positive organizational scholarship (POS), positive institutions, transfer of training, positive design, nudging

## Abstract

Research indicates that Positive Psychology Interventions (PPIs) in the work context have a small positive impact on improving desirable work outcomes, and a small to moderate effect on reducing undesirable work outcomes, suggesting that the effects of PPIs are not trivial, but also not large. Whereas this may be related to the difficulty of changing oneself or one’s happiness levels, the relatively small effects of PPIs may also be due to the predominant use of one-off interventions instead of more structural interventions that reflect policy level commitment. Furthermore, since most PPIs tend to focus on the individual, one could question the long-term effectiveness of such interventions, especially when the work environment remains unchanged. In this manuscript, I introduce a typology of PPIs in organizations by distinguishing between the organizational level they target (the individual or group level), and between one-off and structural interventions. I argue that different types of interventions can strengthen each other, and that to make a sustainable contribution to the optimal functioning of workers, PPIs need to comprise a wide variety of one-off and structural interventions targeting both individuals and groups in organizations. Furthermore, I make suggestions for improving the long-term effectiveness of PPIs by drawing on the literature on transfer of training, nudging, and positive design.

## Introduction

Most adults spend a large part of their life working, and their well-being at work accounts for a large part of the variation in their life satisfaction (Judge and Watanabe, 1993). Furthermore, happy employees have been found to perform better than their less happy colleagues (Cropanzano and Wright, 2001). For these reasons, many scholars and practitioners develop and investigate Positive Psychology Interventions (PPIs) that are aimed to enhance worker well-being. PPIs refer to intentional activities or methods (training and coaching, etc.) based on (a) the cultivation of valued subjective experiences, (b) the building of positive individual traits, or (c) the building of positive institutions (Meyers et al., 2013). Examples of well-known PPIs are interventions that try to enhance feelings of gratitude (Davis et al., 2016), optimism (Malouff and Schutte, 2017), or kind behavior (Curry et al., 2018). Whereas the ultimate aim of PPIs is to increase the well-being of an individual or group (Schueller et al., 2014), when considered in an organizational context PPIs may also be applied to indirectly enhance outcomes such as performance, job satisfaction, leadership skills, and work-life balance (Meyers et al., 2013). A recent meta-analysis on PPIs in a wide variety of contexts (Carr et al., 2020) concluded that PPIs have small to medium positive effects on outcomes related to well-being, and small to medium negative effects on outcomes related to ill-being. A systematic review and a meta-analysis on PPIs in the work context pointed out that on average these interventions have a small positive impact on improving desirable work outcomes, and a small to moderate effect on reducing undesirable work outcomes (Meyers et al., 2013; Donaldson et al., 2019b). So even though the effects of PPIs are not trivial, they are also not large. Apart from the difficulty of changing oneself or one’s happiness levels, and maintaining such behavioral changes (Sheldon and Lyubomirsky, 2021), this may be due to the predominant use of interventions that are based on singular constructs (e.g., gratitude or optimism), that are short-term in nature (Kushlev et al., 2017; White et al., 2019), and that are not structurally embedded in the organizational policies. Furthermore, since most PPIs tend to focus on the individual, one could question the long-term effectiveness of such interventions, especially when the (social) work environment remains unchanged.

In this manuscript, I address the role of PPIs in the creation of positive organizations that facilitate optimal functioning of its members. I introduce a typology of four different types of PPIs in organizations by distinguishing between the organizational level they target (the individual or the group level), and between one-off and structural interventions. I argue that different types of interventions can strengthen each other, and that in order to make a sustainable contribution to the optimal functioning of workers, PPIs need to comprise a wide variety of one-off and structural interventions targeting both individuals and groups. Furthermore, I make suggestions for improving the long-term effectiveness of PPIs by drawing on the literature on transfer of training, nudging, and positive design.

## What We Know About the Effectiveness of Positive Psychology Interventions

Several meta-analyses have been conducted to establish the effectiveness of PPIs in a variety of settings. The first meta-analysis (Sin and Lyubomirsky, 2009) was based on 51 studies that tested interventions, therapies, or activities primarily aimed at increasing positive feelings, positive behaviors, or positive cognitions, and that included pre- and post-intervention measures of well-being or depression, as well as a comparison group. This study pointed out that PPIs have a significant positive effect on well-being and a significant negative effect on depressive symptoms. This study also revealed that PPIs are more effective for people who are depressed, older, or highly motivated to improve, and that longer interventions and interventions that are delivered to individuals (versus groups) are more effective.

Bolier et al. (2013) chose to meta-analyse only randomized controlled studies that were explicitly labeled as PPIs. Based on forty articles they reported small but significant effects for both well-being and depression. Whereas the effects of PPIs at a three to 6 months follow-up were still significant for well-being, they were no longer significant for depression. Furthermore, they found that interventions were more effective if they lasted longer, if recruitment was conducted via referral or hospital, if they were delivered on an individual basis, and if the study design was of low quality. They also established that people with psychosocial problems benefited more from PPIs.

Based on a reanalysis of the studies that were previously included in the meta-analyses of Sin and Lyubomirsky (2009) and Bolier et al. (2013); White et al. (2019) found that when small sample size bias was considered, the effect of PPIs on well-being were small but still significant, whereas the effect of PPIs on depression were generally not statistically significant.

The most recent meta-analysis (Carr et al., 2020) broadly viewed PPIs as evidence-based interventions which have the primary aim of increasing well-being and included 347 PPIs involving over 72,000 participants from clinical and non-clinical child and adult populations. The authors concluded that PPIs have small to medium positive effects on well-being, strengths, and quality of life, and small to medium negative effects on depression, anxiety, and stress, that were maintained at 3 months follow-up. Further, they established that effects were stronger for individuals in non-western countries, and with clinical problems, and that longer programs containing multiple PPIs were more effective.

When looking at the effect of PPIs in the organizational context, less information is available. Meyers et al. (2013) conducted a systematic review of PPIs applied in the organizational context. Based on an analysis of fifteen studies they conclude that PPIs consistently enhance employee well-being. The effects on performance were more ambiguous, which according to the authors could have been caused by a limited sample size or shortenings of the interventions due to the pressure to include them into busy working days. The authors explain the ambiguous effects of PPIs on negative emotional states such as anxiety, burnout, depression, and stress by statistical floor effects since employee populations score rather low on these negative constructs.

A recent meta-analysis by Donaldson et al. (2019a) included intervention studies from positive organizational scholarship (POS), positive organizational behavior (POB), and positive organizational psychology literature (POP), comprising interventions targeting psychological capital, job crafting, strengths gratitude, or well-being. Based on 22 studies they found that PPIs had a small positive effect on improving desirable work outcomes, and a small to moderate effect on reducing undesirable work outcomes. Interestingly, whereas previous meta-analyses found that delivery methods on an individual basis (e.g., individual coaching) were more effective in improving well-being (Sin and Lyubomirsky, 2009; Bolier et al., 2013), they observed a larger effect of PPIs that were delivered in groups, even though individual interventions had a stronger effect on decreasing undesirable work outcomes. Furthermore, they found that gratitude and strengths interventions had stronger mean effect sizes than other interventions on desirable outcomes, whereas interventions targeting psychological capital (i.e., hope, optimism, resilience, and self-efficacy (Luthans et al., 2006) had stronger effect sizes on undesirable outcomes.

### The Psychologistic Fallacy of Positive Psychology Interventions

When looking at the meta-analytic studies, it is striking to see that PPIs focus almost exclusively on individual outcomes such as well-being and depression. Even the studies that focused on PPIs in the organizational context hardly included outcomes beyond the individual level. The review by Meyers et al. (2013), which explicitly aimed to cover outcomes at multiple organizational levels (individual, team, department, and organization), only identified one study that investigated changes in an organizational level variable (service delivery rate of primary care practices) (Ruhe et al., 2011) and one study that investigated changes at the group level (group potency and group identification) (Peelle, 2006). The meta-analysis by Donaldson et al. (2019b) included only two studies that contained outcomes at the collective level, referring to team interactions (Spence Laschinger et al., 2012), and (individual level) perceptions of organizational virtues such as forgiveness, trust, integrity, optimism, and compassion (Williams et al., 2016).

The predominant use of PPIs that focus on the individual is in line with research showing that self-initiated and proactive behaviors can have an important influence on the well-being of workers (Parker et al., 2010; Oprea et al., 2019), and with the trend to encourage workers to take responsibility for their own well-being (Neck and Houghton, 2006). In addition, these individual level PPIs may be particularly relevant for self-employed workers or employees who have little contact with their supervisor or co-workers (Nielsen, 2013). However, the limited focus on individual traits and states is not in line with definitions of PPIs, which also refer to the aim of building positive institutions (Meyers et al., 2013), or enhancing the well-being of groups (Parks and Biswas-Diener, 2013). This neglect of broader socio-environmental influences is also known as the “psychologistic fallacy” (Bacharach et al., 2008); by primarily addressing a person’s psyche, the broader social forces that impact upon on individual’s well-being are ignored (Kern et al., 2020). Given the multi-level nature of work organizations that requires an investigation of intervention outcomes at individual, team, and organizational level (Rousseau, 1985), the focus on individual-level dependent variables represents an important shortcoming of the literature on PPIs in organizations.

The scarcity of PPIs that target the group level is also in sharp contrast with studies that point out that positive states, traits, and behaviors also exist at the group level. For example, West et al. (2009) found that positive psychological capacities such as efficacy, optimism and resilience also function at the team level, and that these team capacities are associated with important team outcomes. van Woerkom et al. (2020) argue that the team context has an important influence on whether individuals’ strengths will be noticed and appreciated by others, and ultimately, whether these strengths will be used. They found that collective awareness of the individual strengths that are represented in the team and the coordination of team roles based on these strengths is associated with both individual performance and leader-rated team performance (Meyers et al., 2020). Furthermore, Fortuin et al. (2021) found that the extent to which team members exhibit mood-enhancing, energizing, and uniting behaviors, directed toward other team members contribute to a positive team climate and teamwork engagement.

By incorporating principles from the systems sciences into the design of the intervention, PPIs can consider that individuals cannot be separated from the broader social systems that they are part of Kern et al. (2020). An example of an intervention informed by systems sciences is Appreciative Inquiry (AI), which aims to create high motivation, high spirit, and cooperation among organizational members as well as a positive and appreciative climate (Whitney and Cooperrider, 1998). AI interventions focus the attention on positive change in an organization by collecting stories of organizational successes, developing ideas for a positive future, designing an organization that makes optimal use of available strengths, and setting up action plans for becoming such an organization (Cooperrider and Whitney, 2005).

### Positive Psychology Interventions as Quick Fixes

The meta-analyses also reveal that the classic PPI is relatively short. In the meta-analysis by Sin and Lyubomirsky (2009), only five of the 49 PPIs that targeted well-being, and only five of the 25 PPIs that targeted depression, lasted longer than 12 weeks. Of the 39 interventions that were included by Bolier et al. (2013) the majority (21) comprised 2 weeks or less. The meta-analysis of Carr et al. (2020) was based on interventions with an average of ten sessions over a 6-week period. When looking at studies in the organizational context, in the systematic review by Meyers et al. (2013) only one of the 15 studies lasted for longer than 11 weeks. Of the 22 studies that were included in the meta-analysis by Donaldson et al. (2019b), the majority (12 studies) included interventions that lasted for less than a month.

Further, only very few studies report on the sustainability of PPIs. Based on their review of 40 studies that reported on PPIs targeting adults in real world settings Hone et al. (2015) conclude that less than half of the studies (43%) assessed individual behavior at least 6 months post-intervention, that only one study reported details of current intervention or program status (2.5%), while none of the studies reported on the costs of maintenance. Furthermore, even though three different meta-analyses (Sin and Lyubomirsky, 2009; Bolier et al., 2013; Carr et al., 2020) pointed out that longer interventions are more effective, possibly because they give participants the opportunity to convert the positive activities they are learning into habits (Fredrickson et al., 2008), short PPIs are more popular in organizations, due to their reduced costs and lost working time (Meyers et al., 2013).

The predominant short-term nature of PPIs and the lack of knowledge about the sustainability of their effects might of course be related to practical problems when conducting long-term (experimental) research in organizations. However, it could also suggest that PPIs are often implemented as quick fixes to address problems with worker well-being, instead of considering them as part of integrated culture change processes and a way of ingraining positive practices in the fabric of an organization (Garcea et al., 2009). A more sustainable implementation of a positive psychology approach in organizations has implications for every part of leadership and HR practices. For this reason, Llorens et al. (2013) recommend incorporating PPIs into the general HR practices of the organization, thereby making them “natural procedures”, that can easily be prompted when needed, and to warrant commitment of the whole organization by providing them with information about the expected gains.

For instance, to sustain worker resilience in the long-term a short one-off training is obviously not sufficient but would need to be complemented by the development of caring relationships among managers and employees, enhancing social support, promoting work–life balance practices, the provision of counseling services, and flexible work arrangements that help workers cope with work and non-work demands (Bardoel et al., 2014). Similarly, implementing a strengths workshop that helps workers to identify their strengths and use these more often at work is unlikely to be successful if such a training is not embedded in an organizational climate and accompanying HR practices (Biswas-Diener et al., 2017). A sustainable implementation of a strengths-based approach requires recruitment and selection practices that are informed by knowledge about the strengths of applicants and the strengths that are lacking in the team, and socialization practices that recognize and highlight newcomers’ strengths at the very beginning of the employment relationship (Cable et al., 2013). Further, idiosyncratic deals that individuals negotiate with their team leader and co-workers (Rousseau et al., 2006) allow for opportunities to tailor job content to individual strengths, and strengths-based performance appraisals (Bouskila-Yam and Kluger, 2011) aimed at discovering unique qualities of workers can give them the opportunity to create a job role in which they can use their strengths to contribute to the team task.

Another way to ensure the sustainability of PPIs is to enhance the well-being of workers by targeting interventions at their leaders. Because leaders can promote a culture of support for employees and hold positions at which they can provide important resources to promote worker well-being, these types of interventions may lead to more sustainable effects (van Woerkom et al., 2021). For instance, Kelloway et al. (2013) show that positive leadership behaviors such as praising, helping, or thanking individuals are associated with employee well-being. Avey et al. (2011) showed that when leaders enact the features of psychological capital (i.e., hope, optimism, resilience, and self-esteem), follower positivity and performance were enhanced. However, a potential risk of interventions targeting leaders is that changes in leader behavior do not always trickle down to the subordinate level (Slemp et al., 2021).

## Four Types of Positive Psychology Interventions

By distinguishing between interventions targeting individuals or groups, and between one-off and structural interventions, four different types of PPIs emerge (see Table 1). This Typology of Positive Psychology Interventions in Organizations (TYPPIO) widens the scope of practitioners and scholars beyond the classical one-off PPI that targets positive states or traits of individuals but let the (social) work environment remain unchanged and alerts them to other types of interventions that address the multi-level nature of work organizations or make use of the opportunity to incorporate interventions into the fabric of the organization (Garcea et al., 2009). Further, this typology enhances awareness of the different roles that different types of intervention may play in enhancing worker well-being. By studying combinations of different types of intervention; individual-level and group-level interventions, and one-off and structural interventions, we can expand our knowledge of the potential of positive psychology to facilitate worker well-being in the long run and add to the building of positive organizations. Below, we discuss examples of each of these four types of interventions, and the role they may play in building positive organizations in a sustainable way.

**TABLE 1:** Types of positive psychology interventions in organizations.

	**Targeting individuals**	**Targeting groups**
One-off	E.g., a 4-week web-based strengths intervention	E.g., a 1-day intervention focusing on appreciative inquiry into best practices and peak experiences in a team
Structural	E.g., implementing a feedforward interview in the performance appraisal procedures	E.g., the implementation of well-being related HR practices

### One-Off Interventions Targeting Individual Level Outcomes

A typical example of a one-off PPI targeting individual outcomes is a 4-week web-based intervention aimed at enhancing the application of signature strengths at work that was investigated by Harzer and Ruch (2016). In this intervention, participants first filled in the Values in Action Inventory of Strengths (VIA-IS; Peterson and Seligman, 2004), and then learned about their four highest character strengths through a web-based training platform. Subsequently, they were asked to think about the ways they currently used their signature strengths in daily activities and tasks at work, and to develop if-then-plans about how to use their four highest character strengths in new and different ways in daily activities and tasks at work. Next, participants were instructed to implement these plans. The authors established that this intervention enhanced the perception of the job as a calling, and satisfaction with life until 6 months after the intervention period.

Even though it is striking that such a relatively light intervention led to effects 6 month later, it is questionable whether these effects can be prolonged if the work environment does not change, and structural interventions that help and remind workers to use their strengths on an ongoing basis are lacking. Further, based on the findings of Seligman et al. (2005) it seems that the novelty-aspect in this exercise is particularly relevant, and that simply displaying more strengths-based behavior does not go along with increases in well-being. This calls into question the effectiveness of prolonging this type of intervention.

However, one-off interventions like this can still play an important role in the creation of positive organizations. First, they may function as an important first step in the transition to a more positive organization by providing workers and leaders with new knowledge and insights based on positive psychology theories, which may in time lead to more structural interventions when they succeed in convincing people of the value of positive psychology. Second, these interventions may indirectly also influence organizational culture, as was shown by Williams et al. (2016) who studied the effects of a 3-day training in psychological capital. Even though this training addressed individual cognitions, they found that respondents with higher levels of PsyCap chose to focus on positive aspects of the organization environment and therefore evaluated the organizational culture more positively. Third, due to hedonic adaptation, workers might over time adapt to the emotional impact of more structural positive organizational practices (Lyubomirsky, 2011), such that they will experience a boost in well-being at the start of the positive change, followed by a return to their original baseline level of well-being as they grow accustomed to the change and begin to take it for granted (Parks et al., 2012). Short one-off interventions that target individual level outcomes may act as boosters of more structural positive interventions that workers might have grown accustomed to.

### Structural Interventions Targeting Individual Level Outcomes

Job redesign that increases the amount of control that employees have over their work is an example of a structural intervention that enhances the well-being of individual workers. For instance, an intervention that gave call center workers more control over their work plan and their development planning, led to stronger perceptions of job control, and an improved mental health (Bond and Bunce, 2001; Bond et al., 2008). Another example of an intervention targeting individual outcomes that could be implemented on a structural basis is a feedforward interview that was implemented as an alternative to the traditional performance appraisal interview (Budworth et al., 2015). For the delivery of this intervention, managers were trained to focus an employee’s attention on a positive work experience involving goal attainment by asking for a specific incident where they felt particularly good about attaining a goal. Furthermore, managers were instructed to ask follow-up questions on the circumstances that enabled their employees to be effective, about the actions that made them feel energized, and about what they could do in coming year to create similar circumstances. This training shifted the role of the manager from judging an employee’s past performance to appreciative inquiry of what an employee will do in the future. The researchers found that the feedforward intervention increased performance 4 months later, relative to the traditional performance appraisal procedure.

Even though the authors of this study unfortunately did not report whether their study in fact led to lasting changes in the performance review practices in the organization, this intervention is a good example of a seemingly effective organizational practice that can replace current structural practices, instead of being implemented as a one-off intervention on top off current practices. This would mean that implementing this intervention does not involve the investment of additional time, apart from a one-time investment in the development of new procedures and training managers and can sustainably be embedded in the organization.

### One-Off Interventions Targeting Group Level Outcomes

Since happiness comes from trying to make others feel good rather than oneself (Titova and Sheldon, 2021), targeting group interactions can be an effective way to enhance well-being in organizations. Furthermore, group interventions can stimulate a social support climate, thereby fostering the commitment and motivation that is needed to sustain intervention effects (Knight et al., 2019). This may especially be the case when employees and employers have a joined responsibility for the design of the intervention, and when the intervention targets leader and employee behaviors as mutually supportive ways to foster employee well-being (see e.g., Kompier et al., 1998; Holman and Axtell, 2016).

An example of an intervention in this category is described in a study by Peelle (2006) among cross-functional teams. The author found that a 1-day intervention focusing on appreciative inquiry into best practices and peak experiences led to higher levels of group identification and in turn group potency, compared to an intervention focusing on creative problem solving. Another example in this category is a 6-month workgroup intervention that aimed at enhancing civility, respect, and engagement in the workplace and in social encounters among people in hospital units (Leiter et al., 2011, 2012). Hospital units were surveyed 3 months before the intervention and unit managers were provided with a profile of their overall results regarding civility, respect, and engagement in comparison with other units for discussion with their staff. Next, all units identified their own goals, agendas, and processes for improving their working relationships in weekly sessions under the guidance of a dedicated facilitator. The intervention resulted in more frequent expressions of appreciation, and in changed workgroup processes, such as including respect as a continuing agenda item on workgroup meetings and improving procedures for registering complaints about mistreatment at work.

### Structural Interventions Targeting Group Level Outcomes

Even though field experiments with structural interventions addressing group level outcomes are uncommon, several studies report that HR practices aimed at enhancing worker well-being have a positive impact on group outcomes. For example, Acosta et al. (2012) established that an organization’s systematic, planned, and proactive efforts to improve employees and organizational health are associated with higher levels of teamwork engagement. These efforts included practices at the task level (e.g., by redesigning tasks to improve autonomy and feedback), social environmental level (e.g., by bidirectional communication to improve social relationships), and organizational level (e.g., by practices that improve the work-family balance). Further, Huettermann and Bruch (2019) found that HR practices aimed at maintaining and promoting employees’ psychological well-being, enhance employees’ collective well-being. More specifically, these HR practices included the prevention of and recovery from work-related psychological health problems, the critical role of top management in communicating the importance of the HR practices, and of leaders in delivering health-related HR practices, and the ongoing evaluation of the HR practices to ensure their effectiveness and sustainability. According to the authors, these practices instilled a positive mindset about the potentially enhancing nature of stress, which in turn, decreased employees’ shared perceptions of how emotionally drained their colleagues are from their work, and increased shared perceptions of how physically, cognitively, and emotionally invested their colleagues are in their work.

Even though future research will have to point out if the aforementioned HR practices have a sustainable effect on group level outcomes, the structural embeddedness of these practices in the organizational procedures make it more likely that their effects will be long lasting.

## Enhancing the Sustainability of Positive Psychology Interventions in Organizations

By investigating a combination of one-off and structural interventions, and interventions that target individual and collective outcomes, we can develop a fuller understanding of the potential of PPIs to contribute to the building of positive organizations. Further, to sustain the effects of PPIs in organizations, researchers and practitioners may benefit from several streams in the literature that so far have been largely neglected in the literature on positive psychology. Below, I discuss some suggestions based on the literature on transfer of training, behavioral change, positive design, and nudging.

### Transfer of Training

The literature on transfer of training signals that training effectiveness in organizational contexts depends on the extent to which the learning that results from a training experience transfers to the job and leads to meaningful changes in work behavior (Blume et al., 2009). Transfer of training is influenced by three primary factors, i.e., learner characteristics, intervention design and delivery, and the work environment. Based on a review of the literature Burke and Hutchins (2007) conclude that cognitive ability, self-efficacy, pre-training motivation, anxiety or negative affectivity, openness to experience, perceived utility of the training, having specific plans for achieving career related goals, and organizational commitment are learner characteristics that are associated with transfer of training. Regarding the intervention design, they found that setting explicit learning goals, a training content that is perceived as relevant to the work task, providing practice and feedback, behavioral modeling (i.e., descriptions of a models’ key behaviors) and error-based examples (i.e., sharing with trainees what can go wrong if they do not use the trained skills back on the job) were relevant factors. Furthermore, they established that supervisory support and peer support are factors in the work environment that enhance transfer. More generally, a transfer climate, referring to cues in the work environment that prompt trainees to use new skills, incentives for correct use of skills, and remediation for not using skills has also been associated with higher chances of transfer. Additionally, they found that transfer is limited when trainees are not provided with opportunities to use new learning in their work setting, for instance because their workload is too high. Other studies on transfer of training suggest that pre-training interventions such as offering attentional advice, preparatory information, and advance organizers, may enhance training effectiveness (Mesmer-Magnus and Viswesvaran, 2010). Also assisting trainees in monitoring their progress toward meeting their objectives or reminding them to continuously answer the question “why am I doing this” may enhance the effectiveness of training (Mesmer-Magnus and Viswesvaran, 2010). Further, post-training interventions such as goal-setting and self-management (Gist et al., 1991; Werner et al., 1994) have proved to improve learning outcomes.

The extensive research on the factors that play a role in transfer of training is particularly relevant to the design of one-off PPIs that are delivered in the form of a training or coaching program. Whereas knowledge about trait-level characteristics of participants (e.g., openness to experience) associated with transfer of training may be more difficult to translate to PPI design, the knowledge about state-level participant characteristics, as well as features of the intervention design and the work environment that contribute to transfer of training can help to make the design of PPIs stronger and embed one-off interventions more structurally in the work environment. For example, the web-based strengths intervention that was developed by Harzer and Ruch (2016) could be strengthened by organizing supervisor or peer support for strengths use or providing a post-training intervention on goal-setting and self-management.

### Positive Design

Another stream in the literature that could be helpful in enhancing the sustainability of PPIs in organizations is the literature on positive design. This literature proposes that interventions do not only include trainings, coaching programs, or apps, but also new organizational policies or practices, or changes in the physical environment (Desmet, 2021). Desmet (2021) identifies four design strategies that can be used to enhance the effectiveness of PPIs by appealing to the needs of the user, and motivating them to adhere to the intervention, i.e., empathic design, participative design, gamification, and persuasive design. Empathic design refers to putting oneself in the position of the target group, their experiences, and latent needs (Koskinen et al., 2003), for instance by meeting the users of the intervention and visiting the places where they will make use of the intervention. Co-design is a strategy in which designers and end-users cooperate to come up with a good design and is based on the idea that end-users of the intervention are experts of their own experiences and therefore have can a unique contribution in the design process. Gamification refers to enhancing motivation and engagement by applying gaming principles in non-gaming environments. For instance, by introducing rewards, making progression visible, and facilitating social interaction, specific activities can be made more challenging and fun, thereby accommodating basic needs for affirmation, growth, autonomy, and self-expression. Persuasive design tries to change attitudes or behavioral patterns of end users by forcing or seducing them into a specific behavior, for example by transferring knowledge, making desired behavior easier, reminding people of intended behavior, activating their norms, using social competition and rewards, or giving immediate feedback (Kelders, 2012).

By applying empathic design, participative design, gamification, and persuasive design PPIs can become more structurally embedded in the physical and social work environment, thereby making their effects more sustainable. Because habits are often strongly connected to the environments in which they occur, changing the environment in cooperation with workers can be a very effective intervention to support behavioral change. For instance, the effect of a 1-day intervention focusing on appreciative inquiry into best practices and peak experiences among cross-functional teams (Peelle, 2006) could be strengthened by designing a room where these teams can meet and engage in fun and relaxing activities during breaks, or by implementing a game that rewards expressing appreciation to other team members.

### Nudging

Another helpful stream in the literature related to persuasive design is that on nudging. The concept of nudging is based on research that shows that many behaviors are directed by unconscious processes and that decisions are often not made based on rational thinking processes, but on quick and automatic heuristic processing (Kahneman, 2011) that is influenced by cues in the environment that people are unaware of. Nudging interventions gently suggest a specific choice by rearranging the choice context (Marchiori et al., 2017), without restricting alternative options or changing financial incentives (Thaler and Sunstein, 2008). Typical nudges change the physical environment or standard options, provide the possibility to correct impulsive choices, or provide feedback on choices (Thaler and Sunstein, 2008).

Several studies investigated the effectiveness of nudging in organizational contexts. One study found that placing a sit-stand desk by default at standing height increased stand-up working rates by approximately seven times (Venema et al., 2018). Other studies found that changing default printer settings to double-sided printing led to significant reductions in paper waste (Egebark and Ekström, 2016), and that desk-based electricity use can be reduced by programming devices to automatically switch off after a period of non-activity (Staddon et al., 2016). A nudge that informed people that an increasing number of people is switching from to-go cups to sustainable alternatives led to a reduced use of disposable cups at work (Loschelder et al., 2019). Making desirable behavior more visible can also be an effective nudge, as was shown by a study that found that medical staff engaged more often in hand hygiene behaviors when they were openly observed (Wu et al., 2018).

Whereas nudges tend not to be effective when people have a strong preference for the alternative option, they are most effective in situations where people are indifferent to the behavior at hand, have good intentions that they forget about, experience conflicting preferences, or do not know what to do because the situation is new to them (Venema and van Gestel, 2021). All these situations may apply after workers have participated in a one-off PPI and may intent to display certain behavior (e.g., focusing on the positive, using their strengths, etc.) but are still unfamiliar with this behavior, forget about it in the madness of the working day, or are being confronted with negative experiences at work. By changing the choice architecture at the workplace, nudging could be a valuable tool for supporting behavioral change that could be incorporated in the design of PPIs. For instance, the effect of a 6-month workgroup intervention aimed at enhancing civility, respect, and engagement in the workplace (Leiter et al., 2011, 2012) could be prolonged by making expressing gratitude to co-workers by default the first point on the agenda of team meetings, or by incorporating feedback on strengths in performance review procedures. Also, the concept of nudging could be used to scrutinize the current choice environment at the workplace for unintended counterproductive nudges in terms of the defaults that are in place and the norms that are being conveyed (Venema and van Gestel, 2021).

## Conclusion

Research on PPIs has greatly contributed to the field of positive psychology in general, and to the field of positive organizational psychology in particular, especially because these studies are generally based on strong, experimental or quasi-experimental designs. However, despite definitions of PPIs that mention the aim of building positive institutions (Meyers et al., 2013), or enhancing the well-being of groups (Parks and Biswas-Diener, 2013), these studies are largely based on interventions that target positive states and traits of individuals but let the (social) work environment remain unchanged. Given the multi-level nature of work organizations, and the importance of positive states, traits, and behaviors at the group level this represents an important shortcoming of the literature. Another drawback of the literature on PPIs in organizations is that it is based on relative short, one-off interventions and that very little is known about the sustainability of their effects (Hone et al., 2015). This could indicate that PPIs are often implemented as quick fixes to problems with worker well-being, instead of as part of integrated culture change processes that are ingrained in the fabric of the organization (Garcea et al., 2009).

In this manuscript, I distinguished between four different types of interventions that can all play a role in the building of positive organizations, i.e., one-off interventions targeting individuals, one-off interventions targeting groups, structural interventions targeting individuals, and structural interventions targeting groups. One-off interventions can play an important role in the beginning of a change process when workers need to be informed about the value of positive psychology. However, structural interventions in the form of HR or leadership practices that are based on the principles of positive psychology are needed to promote, increase, and improve the well-being of all employees on the long term (Paul and Garg, 2014). When these structural interventions are in place, one-off interventions can again strengthen their effect by acting as a booster when the structural attention for specific positive practices in the organizations has begun to wane. Furthermore, one-off interventions can be embedded more structurally in the organization by incorporating insights from research on transfer of training, positive design, and nudging in their design. Whereas structural PPIs that target the group or organizational level are more difficult to assess with (quasi) experimental designs, the effectiveness of these interventions can still be evaluated with a wide array of methods, e.g., by conducting interviews with stakeholders, recording field study notes from workshops, and obtaining experience sampling or diary data (Nielsen and Abildgaard, 2013).

However, if organizations are to embed the principles of positive psychology on a more structural basis in their HR and leadership practices, this will require a shift from a focus on managing employee deficits to a focus on their strengths (Garcea et al., 2009). HR should move to a new phase and start to promote, increase, and improve the well-being of all workers on the long term, and not just workers who are sick, distressed or underperforming (Schaufeli and Salanova, 2010).

## Author Contributions

MW was responsible for the entire article and approved it for publication.

## Conflict of Interest

The author declares that the research was conducted in the absence of any commercial or financial relationships that could be construed as a potential conflict of interest.

## Publisher’s Note

All claims expressed in this article are solely those of the authors and do not necessarily represent those of their affiliated organizations, or those of the publisher, the editors and the reviewers. Any product that may be evaluated in this article, or claim that may be made by its manufacturer, is not guaranteed or endorsed by the publisher.
